# Active Pharmacovigilance for Primaquine Radical Cure of *Plasmodium vivax* Malaria in Odisha, India

**DOI:** 10.4269/ajtmh.21-0816

**Published:** 2022-01-10

**Authors:** Anupkumar R. Anvikar, Prajyoti Sahu, Madan M. Pradhan, Supriya Sharma, Naseem Ahmed, Chander P. Yadav, Sreya Pradhan, Stephan Duparc, Penny Grewal Daumerie, Neena Valecha

**Affiliations:** ^1^ National Institute of Malaria Research, Dwarka, New Delhi, India;; ^2^ National Vector Borne Disease Control Programme (NVBDCP), Odisha, India;; ^3^ Medicines for Malaria Venture, Geneva, Switzerland

## Abstract

*Plasmodium vivax* malaria elimination requires radical cure with chloroquine/primaquine. However, primaquine causes hemolysis in glucose-6-phosphate dehydrogenase-deficient (G6PDd) individuals. Between February 2016 and July 2017 in Odisha State, India, a prospective, observational, active pharmacovigilance study assessed the hematologic safety of directly observed 25 mg/kg chloroquine over 3 days plus primaquine 0.25 mg/kg/day for 14 days in 100 *P. vivax* patients (≥ 1 year old) with hemoglobin (Hb) ≥ 7 g/dL. Pretreatment G6PDd screening was not done, but patients were advised on hemolysis signs and symptoms using a visual aid. For evaluable patients, the mean absolute change in Hb between day 0 and day 7 was −0.62 g/dL (95% confidence interval [CI]: −0.93, −0.31) for males (*N* = 53) versus −0.24 g/dL (95%CI: −0.59, 0.10) for females (*N* = 45; *P* = 0.034). Hemoglobin declines ≥ 3 g/dL occurred in 5/99 (5.1%) patients (three males, two females); none had concurrent clinical symptoms of hemolysis. Based on G6PD qualitative testing after study completion, three had a G6PD-normal phenotype, one female was confirmed by genotyping as G6PDd heterozygous, and one male had an unknown phenotype. A G6PDd prevalence survey was conducted between August 2017 and March 2018 in the same region using qualitative G6PD testing, confirmed by genotyping. G6PDd prevalence was 12.0% (14/117) in tribal versus 3.1% (16/509) in nontribal populations, with G6PD Orissa identified in 29/30 (96.7%) of G6PDd samples. Following chloroquine/primaquine, notable Hb declines were observed in this population that were not recognized by patients based on clinical signs and symptoms.

## INTRODUCTION

Nearly 1.3 billion people are at risk of malaria in India.^1^ The Indian Government has committed to achieve malaria elimination by 2030, developing the National Framework for Malaria Elimination (2016–2030).^2^^,^^3^ Although India has the highest malaria burden in South East Asia, significant progress has been made, with India exceeding its 2020 milestone of a 40% decrease in incidence and mortality compared with 2015.^1^ However, there were still an estimated 5.5 million malaria cases in 2019, and more than half of these were caused by *Plasmodium vivax*.^1^

*Plasmodium vivax* malaria elimination presents specific challenges.^4^ Unlike *Plasmodium falciparum*, *P. vivax* has a dormant hypnozoite liver stage. Hypnozoites are carried asymptomatically, allowing the parasite to persist undetected and sheltered from malaria control efforts.^4^ Hypnozoite reactivation leads to repeated clinical malaria episodes (relapses), which may occur months after the initial infectious inoculation. Relapses also contribute to transmission, leading to outbreaks or transmission reestablishment in areas where malaria has been eliminated.^4^ Prevention of *P. vivax* malaria relapse requires 8-aminoquinoline administration.^4^ However, the 8-aminoquinolines are associated with acute hemolytic anemia in individuals with glucose-6-phosphate dehydrogenase deficiency (G6PDd).^5^^–^^9^

G6PDd is an X-linked enzymopathy prevalent in populations currently or historically exposed to malaria.^10^^–^^12^ With limited metabolic machinery, erythrocytes are wholly dependent upon G6PD to maintain reduced glutathione for protection against oxidative damage.^12^ Oxidative stress can be induced by a number of drugs, including the 8-aminoquinolines. In G6PDd individuals, oxidative damage causes intravascular hemolysis, which may be severe, inducing acute hemolytic anemia.^12^ Being X-linked, G6PDd genotypes are more common in hemizygous males than homozygous females. In heterozygous females, Lyonization results in varied expression of the G6PDd enzyme versus G6PD-normal erythrocytes, with individuals having enzyme activity ranging between that of hemizygous males to normal females.^6^^,^^13^^,^^14^

The G6PD enzyme is highly polymorphic, with deficient variants associated with different degrees of reduced enzyme stability.^12^ Young red blood cells have high G6PD enzyme activity, which declines more rapidly in individuals with G6PDd enzyme variants versus G6PD-normal individuals, and more quickly with “severe” variants (e.g., G6PD Mediterranean) versus those that confer “mild-to-moderate” G6PD deficiency (e.g., G6PD A−, G6PD Mahidol).

The India National Malaria Treatment Guidelines require that confirmed *P. vivax* cases are treated with chloroquine (25 mg/kg over 3 days) and supervised primaquine (0.25 mg/kg/day for 14 days), except for infants under 1 year old, pregnant and lactating women, and individuals known to be G6PDd.^15^ If available, G6PD testing is conducted before primaquine administration. Where G6PD testing cannot be accessed, primaquine is still given, and patients instructed to stop treatment should they experience symptoms of hemolysis, that is, dark urine, weakness, confusion, dizziness, breathlessness, or pale skin.

The State of Odisha is the most malaria-endemic state in India, with 46 million people at risk of infection.^16^^–^^19^ The region is highly receptive to malaria—around a third of Odisha is forested, with mountainous uplands and plateau regions, multiple river systems, associated floodplains, and high rainfall.^20^^,^^21^ Although *P. falciparum* is predominant in Odisha, around 10% of cases are *P. vivax* and the proportion of *P. vivax* tends to increase in areas approaching malaria elimination.^16^ The most common G6PDd genetic variant in the region is G6PD Orissa, considered to have a moderate effect on G6PD enzyme activity (about 10% of normal enzyme activity).^22^^–^^26^ The deployment of *P. vivax* malaria radical cure is complicated by heterogeneity in G6PDd prevalence across the state, being on average around 3%,^23^ but more common in tribal populations, and varying between different tribes from 6% to 17%.^24^^,^^25^^,^^27^^–^^30^ Indigenous tribal people (scheduled tribes) constitute 23% of the population and scheduled castes 17%. Tribal populations generally live in remote villages in hard-to-reach areas with limited access to healthcare.^17^^,^^31^

To improve access to malaria diagnosis and treatment in Odisha, an operational research initiative, the Comprehensive Case Management Project (CCMP), was launched in 2013.^17^^,^^32^ One initiative within the CCMP framework examined primaquine hematologic safety by training Accredited Social Health Activists (ASHAs) to recognize and report hemolytic symptoms following treatment. This approach did not identify any evidence of clinical hemolysis (personal communication Anupkumar R. Anvikar). However, further objective assessment is needed to confirm primaquine hemolytic safety in Odisha under current operational conditions.

This study evaluated the hematologic safety of directly observed 14-day primaquine in *P. vivax* malaria patients in Odisha by measuring hemoglobin (Hb) levels. Pretreatment G6PD testing is not generally available in the region, and point-of-care G6PD kits are not deployed. To reflect usual practice, patients in this study were not screened for G6PDd before primaquine administration. However, to analyze primaquine hematologic safety, qualitative G6PD testing was conducted following study completion. Additionally, G6PDd prevalence in the region was surveyed. The aim was to assess the safety of current practice, that is, using clinical signs and symptoms to identify hemolysis following chloroquine/primaquine in relation to the prevalence of G6PDd in the region. The results will inform policy on implementing well-tolerated and effective universal *P. vivax* radical cure with chloroquine/primaquine to support malaria elimination.

## METHODS

### Design and ethics.

A prospective observational active pharmacovigilance study was conducted between February 2016 and July 2017 in Odisha State, India, at five primary health centers: Paiksahi and Thakurgarh (Angul district), Rasol and Hindol (Dhenkanal district), and Saranggarh (Kandhamal district) within the CCMP (Supplemental Materials; Supplemental Figure 1). Following completion of the observational study, a G6PD prevalence survey (phenotype and genotype) was carried out in the same areas between August 2017 and March 2018. Both studies were conducted according to the principles of the Declaration of Helsinki and Good Clinical Practice and conformed to local and international laws on the study of human subjects. The studies were approved by the Institutional Ethics Committee of National Institute of Malaria Research. Before enrollment to either component, all participants or their parents/guardians completed informed written consent, and school-aged children also had to provide their assent.

### Prospective observational study.

#### Patients.

ASHAs routinely diagnose *P. vivax* malaria based on the results of bivalent rapid diagnostic tests (RDTs) used according to manufacturer’s instructions, in line with national guidelines. Deployed RDTs were SD Bioline Malaria Ag Pf/Pv (Abbott, New Delhi, India), EzDx™ Malaria Pv/Pf Ag (Advy Chemical, Thane, India), and Onsite Malaria Pf/Pv Ag (CTK Biotech Inc, New Delhi, India). Eligible patients were at least 1 year old with RDT-confirmed *P. vivax* malaria, Hb of at least 7 g/dL assessed using HemoCue^®^ point-of-care testing (HemoCue India, New Delhi, India), and willing and able to participate in the study. The ASHAs referred patients to study sub-centers for Hb assessments and therapy initiation.

#### Treatment.

Patients were treated in accordance with national antimalarial drug policy, dosed by body weight with 25 mg/kg chloroquine over 3 days plus primaquine 0.25 mg/kg per day for 14 days. All treatment was directly observed by ASHAs or healthcare workers. All patients were advised of the signs and symptoms of hemolysis and the need to seek medical advice in alignment with CCMP protocols.

#### Procedures.

Following enrollment, all patients underwent a physical examination and had a medical history taken. At the study sub-center, before treatment initiation, duplicate baseline Giemsa-stained blood slides were prepared for confirmation of malaria diagnosis by two trained microscopists using standard methods.^33^ Parasitemia was also assessed at day 3, and should malaria symptoms recur.

Patients were followed up daily during their 14-day primaquine course by ASHAs. ASHAs were compensated (Rs 150) by the NVBDCP/National Health Mission for each *P. vivax* case who completed treatment. Patients were asked to report symptoms of acute hemolytic anemia, that is, excessive tiredness, dizziness, breathlessness, dark-colored urine, or pale skin. ASHAs used a visual instruction tool to educate and question patients regarding any notable symptoms (Supplemental Figure 2). If any symptoms were identified, the patient was referred to the nearest primary health center for assessment and management.

Hemoglobin levels were measured at baseline (day 0) and days 3, 7, 14, 28, and 42 using HemoCue^®^. Anemia was defined using World Health Organization criteria.^34^ Any patient with an Hb decrease of ≥ 2 to < 3 g/dL from baseline was followed up daily in the community until Hb levels normalized. An Hb decline ≥ 3 g/dL prompted referral to hospital for investigation and treatment if clinically indicated.

Following study completion, G6PD qualitative testing was conducted to inform the analysis of Hb levels. Approximately 3 mL blood was collected in an ethylenediaminetetraacetic acid (EDTA) Vacutainer from each subject and G6PD phenotyping was performed within 24 hours of sample collection using the dichlorophenol indophenol (DCIP) dye discoloration test as per published methods.^35^ If qualitative testing indicated that a sample was G6PDd, confirmatory G6PD genotyping was conducted using published methods at a nationally accredited laboratory (for more details, see G6PD prevalence study below).^36^^,^^37^

#### Outcomes and statistics.

The primary study endpoint was the absolute and proportionate fall in Hb from day 0 to day 7, and Hb recovery from day 28 to day 42. The number and proportion of patients who had adverse events, required hospitalization, underwent blood transfusion, or had treatment discontinued, were also evaluated. Data were presented using descriptive statistics. Statistical comparisons used the Mann–Whitney test. Statistical analysis was performed in GraphPad Prism (version 9.0).

#### Sample size.

Assuming a prevalence of fall in Hb among *P. vivax* cases of 90%, with 5% absolute precision and 95% confidence intervals (CIs), 138 patients with *P. vivax* malaria were needed to estimate the prevalence of Hb declines.

### G6PDd prevalence survey.

#### Subjects and sample size.

A cluster sampling method was followed with villages in the catchment areas of the pharmacovigilance study chosen at random. Study enrollment was offered to all individuals in the sampled villages. All subjects who provided written informed consent were included in the study. Assuming an average prevalence of G6PDd of 10%, relative precision of 20%, and desired CI (1 alpha) of 90%, in a population of 300,000, a sample size of 604 subjects was required. Statistical comparisons used the Mann–Whitney test (GraphPad Prism, version 9.0).

#### Procedures.

A 3 mL-blood sample was collected in an EDTA Vacutainer from each subject for G6PD phenotyping using the DCIP test as described above.^35^ Samples identified as G6PDd based on qualitative testing were further evaluated with G6PD quantitative testing and G6PD genotyping at a central laboratory (Dr Lal Path Laboratories, Gurgaon, India). Quantitative G6PD phenotyping used a World Health Organization standardized method (G6PD assay of hemolysates: normal reference range 7.0–20.5 IU/gHb).^36^ A blood spot was collected on filter paper for G6PD genotyping, performed according to published methods.^37^ Briefly, genomic DNA was isolated using QIAamp DNA Blood Mini Kit (Cat No- 51104, Qiagen, Hilden, Germany) before amplification of the G6PD exon and DNA sequencing.^37^ Specific primers were used to amplify the most commonly identified G6PDd genetic mutations in India and Odisha, that is, G6PD Orissa (C131G), G6PD Mediterranean (C563T), and G6PD Kerala-Kalyan (G949A).^38^^–^^40^ Amplification products were checked on 1% Agarose gel before undergoing restriction fragment length polymorphism analysis using restriction enzymes MboII for G6PD Mediterranean, Hae III for G6PD Orissa, and MnlI for G6PD Kerala-Kalyan.^38^

## RESULTS

### Prospective observational study.

#### *Plasmodium vivax* patients.

Of the 100 patients enrolled, 54% were over 14 years old and 61% had baseline mild/moderate anemia (Table 1).^34^ Most patients were from Angul (Thakurgarh *N* = 42, Paiksahi *N* = 18), followed by Dhenkanal (Hindol *N* = 10, Rasol *N* = 14) and Kandhamal (Saranggarh *N* = 16). The population from Kandhamal differed from the other regions, comprising all males over 14 years old. Two patients had missing day 7 values (one male, one female, both from Angul). One patient was lost to follow up with no post-baseline evaluations. Overall, 11 patients were lost to follow up (four females, seven males, all from Angul), with 89% (89/100) of patients having baseline, day 28 and day 42 Hb values. Although efficacy was not formally evaluated in this study, there were no cases of recurrent malaria infection reported over the 42-day follow up.

**Table 1:** Patient baseline demographic and clinical characteristics

Characteristic	Total (*N* = 100)	Angul (*N* = 60)	Dhenkanal (*N* = 24)	Kandhamal (*N* = 16)
Sex				
Males, *n*(%)	54 (54.0)	27 (45.0)	11 (45.8)	16 (100)
Females, *n*(%)	46 (46.0)	33 (55.0)	13 (54.2)	0
Nontribal, *n*(%)	60 (60.0)	41 (68.3)	18 (75.0)	1 (6.3)
Tribal	40 (40.0)	19 (31.7)	6 (25.0)	15 (93.8)
Mean age (SD), years	20.7 (16.3)	20.0 (15.9)	21.7 (20.6)	22.0 (10.0)
Number (%) of patients aged				
1 to ≤ 5 years	13 (13.0)	9 (15.0)	6 (25.0)	0
> 5 to ≤ 14 years	33 (33.0)	24 (40.0)	7 (29.2)	0
> 14 years	54 (54.0)	27 (45.0)	11 (45.8)	16 (100)
Mean (SD) baseline hemoglobin, g/dL				
Males	12.3 (2.1)	11.3 (1.9)	12.2 (1.5)	14.0 (1.6)
Females	11.0 (1.1)	10.9 (1.2)	11.3 (0.9)	–
Anemia, *n*(%)*				
None	39 (39.0)	16 (26.7)	12 (50.0)	11 (68.8)
Mild	35 (35.0)	22 (36.7)	8 (33.3)	5 (31.3)
Moderate	26 (26.0)	22 (36.7)	4 (16.7)	0

* Based on World Health Organization criteria.^34^

Qualitative G6PD testing, conducted following study completion, indicated that 68.5% (37/54) of male patients were G6PD normal, G6PD enzyme activity status was unknown for the remaining 17 patients with no known G6PDd patients. In females, 87.0% (40/46) had normal G6PD enzyme activity based on qualitative testing, G6DP status was unknown for four patients, and there were two G6PDd female patients, both of which were confirmed by genotyping as heterozygous for G6PD Orissa.

#### Hematologic safety.

For the primary endpoint, the mean absolute change in Hb between day 0 and day 7 was significantly higher for males (−0.62 g/dL [95%CI: −0.93, −0.31] *N* = 53) versus females (−0.24 g/dL [95%CI: −0.59, 0.10] *N* = 45; *P* = 0.034) (Table 2). However, the mean percentage change in Hb between day 0 and day 7 was not significantly different between males (−4.46% [95%CI: −7.27, −1.65]) and females (−1.71% [95%CI: −4.97, 1.54] *P* = 0.093) (Table 2). In males, the mean absolute decline in Hb from day 0 to day 7 was more pronounced in tribal versus nontribal patients (*P* = 0.0097), as was the mean percentage decline in Hb (*P* = 0.018) (Table 2). In contrast, in females, the mean absolute change and percentage change in Hb from day 0 to day 7 was similar in tribal versus nontribal patients (*P* > 0.47) (Table 2).

**Table 2:** Mean absolute and percentage changes in hemoglobin (Hb) from baseline (day 0) to day 7 in *Plasmodium vivax* patients following chloroquine/primaquine administration

Patient group	Mean absolute change in Hb (95%CI)	Percentage change in Hb (95%CI)
Males (*N* = 53)	−0.62 (−0.93, −0.31)	−4.46 (−7.27, −1.65)
Females (*N* = 45)	−0.24 (−0.59, 0.10)	−1.71 (−4.97, 1.54)
Comparison	*P* = 0.034	*P* = 0.093
Nontribal males (*N* = 26)	−0.27 (−0.71, 0.18)	−1.61 (−5.49, 2.27)
Tribal males (*N* = 27)	−0.97 (−1.38, −0.55)	−7.21 (−11.22, −3.20)
Comparison	*P* = 0.0097	*P* = 0.018
Non-tribal females (*N* = 32)	−0.18 (−0.66, 0.29)	−0.95 (−5.38, 3.47)
Tribal females (*N* = 13)	−0.39 (−0.77, −0.00017)	−3.56 (−7.17, −0.00016)
Comparison	*P* = 0.52	*P* = 0.47

CI = confidence interval. Exact *P* values were calculated using the Mann–Whitney test.

**Figure 1.**
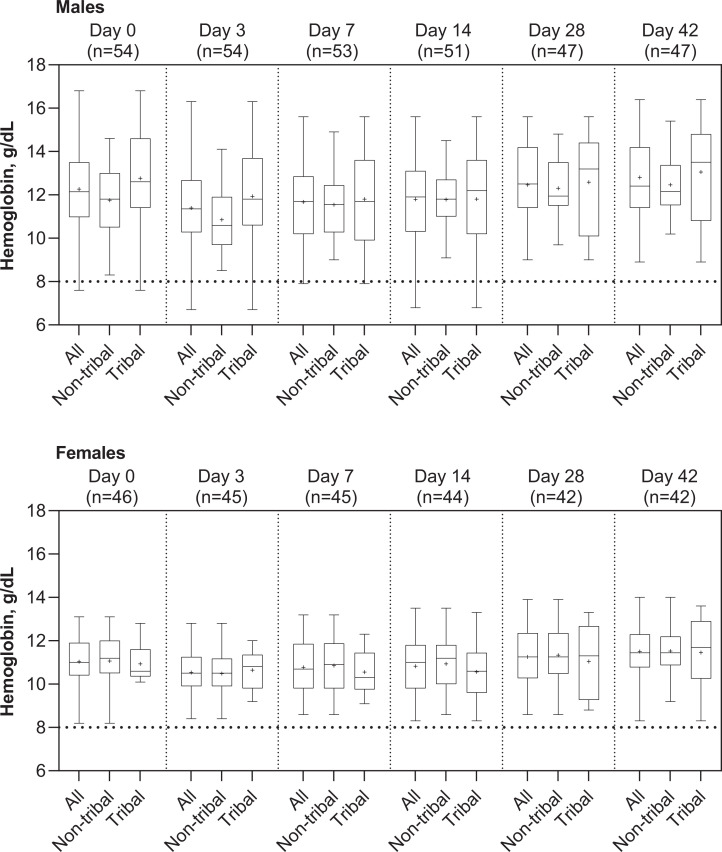
Hemoglobin levels in male and female *Plasmodium vivax* malaria patients at baseline (day 0) and following 3-day chloroquine plus 14-day primaquine. Means are shown as +, boxes are the median with 25th and 75th percentile bounds and whiskers are maximum and minimum values.

The mean maximum absolute change from baseline in Hb in males (*N* = 54) was −1.4 g/dL (95%CI: −1.7, −1.1; range: −4.7 to 1.0 g/dL) and in females (*N* = 45) was −0.97 g/dL (95%CI: −1.3, −0.64; range: −4.1 to 2.2 g/dL) (*P* = 0.062) (Figure 1). The mean maximum percentage change from baseline in Hb in males was −11.1% (95%CI: −13.4, −8.8) and −8.3% (95%CI: −11.3, −5.4) in females (*P* = 0.18) (Figure 1). In those patients with a baseline Hb <11.5 g/dL, 77.6% (38/49) had Hb declines to below their baseline Hb during the first 7 days compared with 95.9% (47/49) of those with a baseline Hb ≥ 11.5 g/dL.

By day 28, Hb had recovered to at least baseline levels in 61.7% (29/47) of males and 54.8% (23/42) of females, and by day 42, 70.2% (33/47) of males and 59.5% (25/42) of females had Hb levels equivalent or higher than at baseline (Supplemental Figure 3). At day 42, 31.5% (28/89) of patients had mild anemia and 16.9% (15/89) had moderate anemia, but none had severe anemia.

A post hoc analysis considered only those patients with a post-baseline Hb decline (*N* = 89). In these patients, the Hb nadir occurred most commonly at day 3 (*N* = 33) with a mean Hb decline of −1.3 g/dL (95%CI: −1.6, −1.0; range: −2.8 to −0.1 g/dL). Hb declines were most pronounced on day 14 (*N* = 20) with a mean Hb decline of −1.8 g/dL (95%CI: −2.3, −1.2; range: −4.7 to −0.1 g/dL) (*P* = 0.5) (Supplemental Figure 4). The nadir for Hb declines ≥ 3 g/dL only occurred on days 7 (*N* = 1) and 14 (*N* = 4).

**Figure 2.**
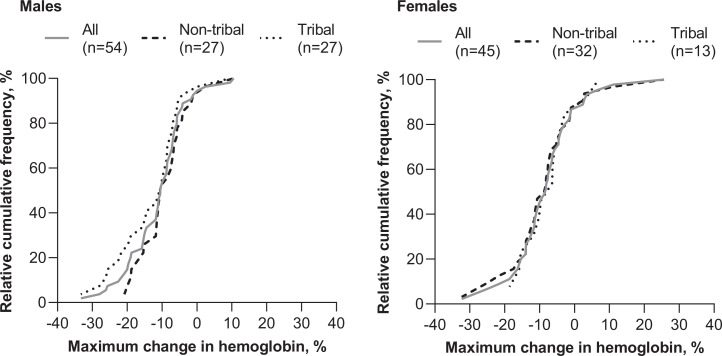
Effect of tribal status on hemoglobin (Hb) levels in male and female *Plasmodium vivax* malaria patients following 3-day chloroquine plus 14-day primaquine. Ogive plot of the maximum percentage change in Hb from baseline (day 0) at any study time point.

An Hb decrease of ≥ 10% was noted for 53.7% (29/54) of males and 44.4% (20/45) of females (Figure 2). Hemoglobin declines ≥ 20% were more common in tribal males (22.2% [6/27]) than in nontribal males (7.4% [2/27]) (Figure 2). For females, Hb declines ≥ 20% were less common in tribal (0% [0/13]) versus nontribal females (12.5% [4/32]) (Figure 2).

Declines of Hb ≥ 2 g/dL versus baseline occurred in 19.2% (19/99) of patients. Analysis of the relationship to G6PD phenotype determined at study completion showed that these patients included one G6PDd female, 13 patients with G6PD-normal phenotype (10 males, three females), and five patients with unknown G6PD status (four males, one female) (Supplemental Figure 5).

There were six patients with an absolute Hb value < 7 g/dL and/or Hb decreases ≥ 3 g/dL. Two patients had Hb values < 7 g/L, both were from Angul, one 4-year-old male (G6PD-normal phenotype) had a baseline Hb of value of 7.6 g/Hb, declining to 6.7 g/dL on day 3 and 6.8 g/dL on day 14, with recovery to 11.4 g/dL on day 28. One 37-year-old male (G6PD phenotype unknown) had a baseline Hb value of 10.2 g/dL, declining to 7.9 g/dL on day 7 and of 6.8 g/dL on day 14, with recovery to 10.3 g/dL on day 42. For the five patients with Hb decreases ≥ 3 g/dL, three were tribal males (two G6PD-normal phenotype, one G6PD phenotype unknown) and two were nontribal females (one G6PD-normal phenotype, one G6PDd genotype [confirmed by G6PD genotyping as heterozygous]). This G6PDd heterozygous patient had a baseline Hb level of 12.0 g/dL, a nadir of 9.0 g/dL on day 14, and recovery to 12.3 g/dL by day 42 (Supplemental Figure 5). An additional G6PDd heterozygous female had a maximum Hb decline of −0.3 g/dL from 10.6 g/dL at baseline to 10.3 g/dL on day 7, with recovery to 11.0 g/dL by day 14.

#### Clinical safety.

**Figure 3.**
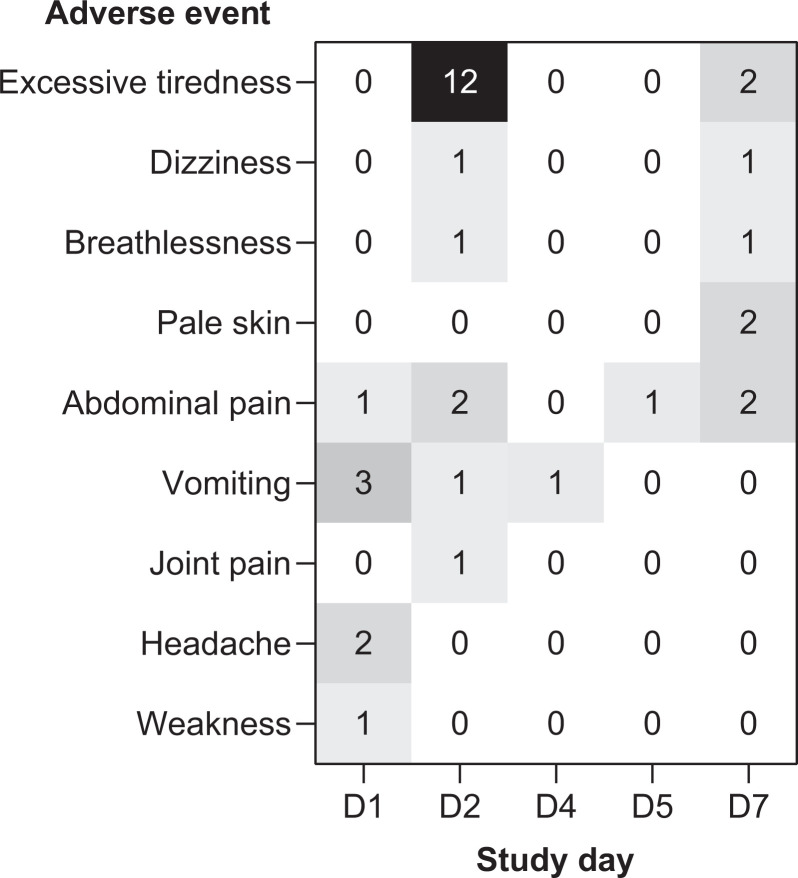
Number of adverse events reported during the observational study. No adverse events were reported on days 3 and 6 and between days 8 and 42 inclusive.

Treatment was generally well tolerated (Figure 3). The majority of adverse events were noted on days 1 and 2. Excessive tiredness was noted in 12 patients on day 2, that is, after only 1 day of primaquine and therefore most probably related to malaria rather than hemolysis. Vomiting on days 1 and 2 was associated with chloroquine administration. There were three hospitalizations, two were associated with declines in Hb and one was associated with chloroquine intolerance that resolved without further treatment. Some patients who met the criteria for hospitalization owing to low Hb refused admission because they were asymptomatic. No patient required blood transfusion. Supportive iron supplements were given by ASHAs to three patients.

For the 94 patients followed up on day 7, eight adverse events occurred in four patients that could potentially indicate hemolytic anemia (Figure 3). However, of these four patients, only two had Hb declines from baseline ≥ 2 g/dL: one 14-year-old female experienced excessive tiredness, breathlessness, and pale skin on day 7 with an Hb decline of −2.8 g/dL on day 3 (Hb level 8.4 g/dL) recovering to −0.2 g/dL versus baseline by day 7 (Hb level 11.0 g/dL); one 4-year-old male had dizziness, pale skin, and abdominal pain on day 7 with a maximum Hb decline on day 7 of −2.2 g/dL (Hb level 9.9 g/dL). Notably, none of the six patients with an Hb drop of ≥ 3 g/dL and/or Hb levels < 7 g/dL complained of the clinical signs or symptoms of acute hemolytic anemia.

### G6PDd prevalence survey.

The G6PDd prevalence study included 626 participants. Based on qualitative testing (DCIP), 5.1% (95%CI: 3.6, 7.1 [32/626]) of participants were G6PDd or G6PD intermediate (Table 3). However, following confirmatory testing, two participants identified as G6PDd with the DCIP test had normal G6PD enzyme levels based on quantitative testing (11.54 and 11.77 IU/gHb) and a G6PD-normal genotype. Thus, based on qualitative testing confirmed by genotyping, 4.8% (95%CI 3.4, 6.8 [30/626]) of participants were G6PDd (Table 3). The G6PD Orissa variant was identified in 96.9% (29/30) of G6PDd samples, plus one unknown G6PDd genotype. Confirmed G6PDd was more common in tribal (12.0%; 95%CI: 7.3, 19.1 [14/114]) versus nontribal participants (3.1%; 95%CI: 1.9, 5.0 [16/509]). However, there were considerable differences in prevalence between individual tribes, though numbers were small and CIs wide.

**Table 3:** Prevalence of G6PD deficiency determined by qualitative testing and confirmed by quantitative testing and G6PD genotyping

Category	*n*/*N*	Percentage (95% CI)
Qualitative testing	32/626	5.1 (3.6, 7.1)
Confirmed by quantitative testing and confirmatory genotyping*	30/626	4.8 (3.4, 6.8)
Gender		
Male	15/243	6.2 (3.8, 9.9)
Female	15/383	3.9 (2.4, 6.4)
Age		
≤ 5 years	2/20	10.0 (1.8, 30.1)
6–14 years	7/169	4.1 (2.0, 8.3)
> 15 years	21/437	4.8 (3.2, 7.2)
District		
Angul	17/198	8.6 (5.4, 13.3)
Angul—males	9/77	11.7 (6.3, 20.7)
Angul—females	10/121	8.3 (4.6, 14.6)
Dhenkanal	10/218	4.6 (2.5, 8.2)
Dhenkanal—males	5/85	5.9 (2.5, 13.0)
Dhenkanal—females	5/133	3.8 (1.6, 8.5)
Kandhamal	3/210	1.4 (0.4, 4.1)
Kandhamal—males	1/81	1.2 (0.1. 6.7)
Kandhamal—females	2/129	1.6 (0.3, 5.5)
Nontribal	16/509	3.1 (1.9, 5.0)
Nontribal males	6/191	3.1 (1.4, 6.7)
Nontribal females	10/318	3.1 (1.7, 5.7)
Tribal	14/117	12.0 (7.3, 19.1)
Tribal males	9/52	17.3 (9.4, 29.7)
Tribal females	5/65	7.7 (3.3, 16.8)
Tribe		
Bantha	1/14	7.1 (0.4, 31.5)
Dehuri	10/68	14.7 (8.2, 25.0)
Majhi	0/14	0 (0, 21.5)
Pingua	2/2	100.0 (17.8. 100)
Punta	1/2	50.0 (2.6, 97.4)
Others	0/17	0.0 (0.0, 18.4)

G6PD = glucose-6-phosphate dehydrogenase.

* Participants were only genotyped if they were found to be G6PDd based on qualitative testing. Genotyping confirmed 30/32 G6PDd cases identified using qualitative testing.

**Figure 4.**
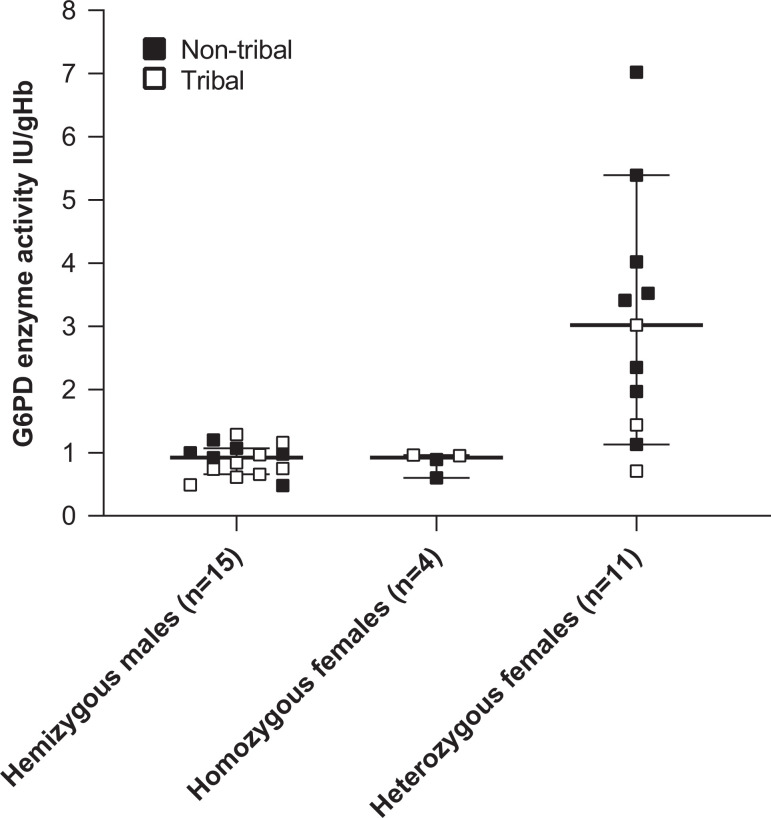
Quantitative G6PD enzyme activity in individual survey participants confirmed by genotyping as G6PDd. Horizontal bars show the median plus or minus the 95% confidence intervals.

In participants with a G6PDd genotype, the median (range) G6PD enzyme level was 0.92 (0.48, 1.29) IU/gHb in males (*N* = 15) versus 1.97 (0.60, 7.02) IU/gHb in females (*N* = 15) (*P* = 0.0075) overall (Figure 4). Median enzyme levels were 0.92 (0.60, 0.96) IU/g/Hb in G6PDd homozygous females (*N* = 4) and 3.0 (0.7, 7.0) IU/gHb in heterozygous females (*N* = 11) (Figure 4). There was no pattern in the degree of G6PD enzyme activity in tribal (*N* = 14) versus nontribal persons (*N* = 16), either between males (*P* = 0.39) or females (*P* = 0.16) (Figure 4).

## DISCUSSION

This study aimed to evaluate whether the currently applied clinical pathway of administering chloroquine/primaquine without prior G6PD testing, but with advice on the signs and symptoms of hemolysis and community follow-up, was able to detect significant declines in Hb. We also surveyed G6PDd prevalence in the population.

*Plasmodium vivax* infection is associated with anemia. Also, following malaria treatment initiation, small declines (< 1 g/dL) in Hb levels, which recover after day 3, are caused by hemodilution on rehydration. Consistent with previous studies,^41^ mean maximum declines in Hb following chloroquine/primaquine were modest, being < 2 g/dL. However, the hematological profiles of some individual patients were concerning. Absolute Hb levels < 7 g/dL Hb and/or declines from baseline ≥ 3 g/dL occurred in six patients. Although none of these patients reported clinical signs of acute hemolytic anemia, hemoglobin drops of this magnitude are of physiological relevance, particularly if they are caused by intravascular hemolysis.^42^ From an operational perspective, these findings indicate that the visual aid and health worker follow-up was an insensitive tool for detecting absolute Hb levels that fell to < 7 g/dL and/or Hb declines ≥ 3 g/dL.

The disparity between clinical and laboratory findings was surprising as patients had been instructed to report all potentially relevant symptoms of hemolysis, even if they were not worsening, and were followed up daily by ASHAs. As 61.0% (61/100) of patients had mild-to-moderate anemia at study entry, it is possible they were adapted to living with low Hb levels and so did not manifest clinical signs of hemolysis. Also, in this area of high malaria endemicity, patients may expect to feel poorly, with common symptoms not appearing noteworthy, and patients may have been less diligent in their observations once their fever resolved at day 2 or 3. Dark-colored urine was not reported by any patient, and this might reflect the available toilet facilities, which make such observations difficult. These findings suggest that our current practice would not be expected to identify cases that might require medical intervention. Moreover, because declines ≥ 3 g/dL occurred at day 7 and day 14 following treatment initiation, patients may be distant from health services.

Of the five patients with Hb decreases ≥ 3 g/dL, one was G6PDd, three had a G6PD-normal phenotype, and one had an unknown G6PD status. Thus, primaquine-induced hemolysis was suspected for at least one patient with a ≥ 3 g/dL Hb decline, and considered as a possible causality for the patient whose G6PD status was unknown. However, we did not expect to see notable Hb declines in three patients with a G6PD-normal phenotype. Because G6PD genotyping was only performed on samples identified as G6PDd following qualitative testing, we cannot exclude that these phenotypically “G6PD-normal” patients were false-negative results. For example, in cases of acute hemolysis associated with malaria, the erythrocyte population is enriched for young erythrocytes and reticulocytes, which have near-normal G6PD enzyme levels.^43^ However, as G6PD qualitative tests were conducted following study completion, it is unlikely that these patients would retain a young enough erythrocyte population to return a false-negative finding. Also, although heterozygous females with intermediate enzyme levels will not be detected using qualitative testing, hemolytic risk is a function of G6PD enzyme activity, rather than genotype. This has been demonstrated in heterozygous females with intermediate G6PD levels for both primaquine and tafenoquine.^6^^,^^8^^,^^14^ Thus, primaquine-induced hemolysis would not be expected if G6PD enzyme activity were normal, even in an individual with a G6PDd genotype. G6PD genotyping of all patients with Hb declines ≥ 3 g/dL would have resolved these uncertainties.

The general health of tribal populations is poorer and socioeconomic status lower than nontribal populations.^44^ This may cause such populations to be more susceptible to malarial anemia and/or other causes of anemia such as malnutrition, and this might explain the greater Hb falls in the tribal population. However, although Hb declines ≥ 20% were more common in tribal males versus nontribal males, there was no difference between tribal females versus nontribal females. Hematologic disorders such as β-thalassemia and sickle cell anemia are more prevalent in tribal populations and may affect males more severely.^22^^,^^30^^,^^45^ However, testing for these hemoglobinopathies was not conducted during this study and would not be routinely available in the study areas.

After 42 days of follow up, recovery to baseline Hb levels had not been achieved in 29.8% of males (14/47) and 40.5% of females (17/42). However, the hematologic consequences of not administering primaquine could not be evaluated in this study. Longer term hematologic benefits and wider health benefits from primaquine therapy versus repeated *P. vivax* relapses have been reported from areas where *P. vivax* has a short-latency relapse periodicity.^41^^,^^46^^–^^51^ However, given the long latency period for relapse in India, the potential to improve longer term hematologic and other outcomes requires evaluation.

The G6PDd survey used a qualitative test, with G6PDd samples confirmed using G6PD quantitative testing and genotyping. The overall G6PDd prevalence was 4.8% (30/626). This is higher than identified in the Kalahandi and Rayagada districts of Odisha state (3.0% [59/1981]),^23^ but similar to that reported in northeastern Indian states (5.4% [55/1015]).^52^ Consistent with previous findings,^23^^,^^24^ our data show that tribal populations had a higher prevalence of G6PDd (12.0%) versus nontribal populations (3.1%), increasing to 17.3% in tribal males.

Consistent with previous studies, G6PD Orissa was the most prevalent G6PDd variant in Odisha, detected in 29/30 of the G6PDd samples confirmed by genotyping.^24^^,^^39^ One sample had intermediate G6PD activity by quantitative testing (11.5 IU/gHb) but had a genotype that was not one of the three G6PD variants assessed (Orissa, Mediterranean, and Kerala-Kalyan). It is a limitation of our study that we cannot exclude the presence of other G6PDd variants in Odisha and more extensive genome sequencing studies are required to determine the range and distribution of different G6PDd variants in the region. For example, in Kalahandi and Rayagada provinces in Odisha, in addition to G6PD Orissa (87.9%), G6PD Kaiping (0.15%), G6PD Coimbra (0.1%), and G6PD Union (0.05%) have been found at low frequencies.^23^

As expected from previous reports using qualitative G6PD testing, the proportion of G6PDd males (6.2%) was higher than that of females (3.9%). Because heterozygous females have a mixture of G6PD-normal and G6PDd erythrocytes, only a proportion of G6PDd heterozygous females will be detected using phenotypic G6PD testing.^6^^,^^13^ Assuming a Hardy–Weinberg equilibrium, the allelic prevalence of the G6PDd gene should be 12.0% in females overall, 6.1% in nontribal females, and 31.6% for tribal females. Thus, 32.5% of all females, 50.8% of nontribal females, and 24.4% of tribal females with G6PDd alleles were detected. Hemolysis in G6PDd heterozygotes has been previously demonstrated following primaquine (G6PD Mahidol),^14^ tafenoquine (G6PD Mahidol),^8^ and with an anti-malarial containing dapsone (G6PD A−).^13^ However, a cut-off for G6PD enzyme activity that can be considered properly tolerated for primaquine administration has not been formally defined.^6^^,^^14^ Thus, an unknown proportion of G6PD-heterozygous females are at risk of acute hemolytic anemia following primaquine-induced oxidative stress and the number of females at risk in our survey will have been underestimated.

There were some key limitations across the two studies. Most notably, because this study was conducted in an operational context to evaluate current practice, there was no chloroquine-only control arm. The efficacy of primaquine versus chloroquine alone at preventing *P. vivax* relapse is well established.^53^ Also, the time course of changes in Hb with primaquine versus chloroquine has been previously documented through both clinical studies and modeling.^41^^,^^54^^,^^55^ Additionally, because sample times for Hb levels were infrequent, we cannot accurately define the time course of Hb changes. The hemolytic effects of primaquine are likely to be most evident from day 3 after dosing.^6^^,^^56^ Thus, by observing Hb at day 7, we are likely to have captured any notable hematologic consequences of primaquine administration. The target sample size for the operational study was 138, but only 100 patients were recruited, reflecting the trend for reduced malaria prevalence in the region. Note that our study was designed to evaluate whether our current methods of patient-reported clinical signs and symptoms of hemolysis could identify Hb declines, and G6PD testing was only done after the study end to inform the interpretation of those results. Thus, the study was not designed or powered to determine the frequency of primaquine-induced hemolysis in G6PDd individuals. A significant limitation of the study is that G6PD genotype was only determined for samples that were G6PDd or G6PD-intermediate based on qualitative phenotyping. Thus, we were unable to estimate the frequency of false-negative results for the DCIP test, though two results were false positives. Also, as hematology and clinical laboratory investigations were not conducted, hemolytic markers, such as haptoglobin, unconjugated bilirubin, and reticulocytes, could not be assessed. These limitations are consistent with the conduct of a real-world, operationally relevant study conducted with resources that are routinely available at the study sites or regional laboratories. Although primaquine hematologic safety is well described,^41^ the current study was designed to inform policy implementation specifically in the population of Odisha.

In summary, notable declines in Hb were observed in some *P. vivax* patients in Odisha following treatment with chloroquine/primaquine. The visual instruction tool plus frequent and specific questioning by ASHAs did not identify patients with Hb declines that might require intervention. Qualitative G6PD testing (DCIP) conducted after study completion did not reliably predict patients’ risk of Hb decline. The declines in Hb observed could be caused by primaquine/chloroquine administration, malaria, or other factors, and they may be multi-factorial. The survey confirmed that G6PDd prevalence was higher in the tribal versus nontribal population.

It is clear that radical cure is a key tool for malaria elimination in Odisha, but clinical observations could not be relied upon to identify patients with physiologically relevant Hb declines. Given the high prevalence of G6PDd alleles in Odisha, particularly in tribal populations, rather than attempting to detect primaquine-induced hemolysis, it would be preferable to avoid exposing those at risk by wider deployment of pretreatment G6PD testing. However, as the DCIP qualitative testing method was inadequate to predict individuals at risk of Hb declines, the utility of advanced point-of-care quantitative diagnostics should be evaluated.^57^ Additional health interventions to address anemia and identify hemoglobinopathies should also be investigated to improve the hematologic safety of radical cure in this population.

## Supplemental Material


Supplemental materials
