# Supplementary material for: Active Pharmacovigilance for Primaquine Radical Cure of Plasmodium vivax Malaria in Odisha, India
Source: Am J Trop Med Hyg. 2022 Jan 10;106(3):831–40. doi: 10.4269/ajtmh.21-0816 (PMC8922502; doi:10.4269/ajtmh.21-0816)

Supplementary materials

Active pharmacovigilance for primaquine radical cure of *Plasmodium vivax* malaria in Odisha, India

Anupkumar R. Anvikar,[1*] Prajyoti Sahu,[2] Madan M. Pradhan,[2] Supriya Sharma,[1] Naseem Ahmed,[1] Chander P. Yadav,[1] Sreya Pradhan,[2] Stephan Duparc,[3] Penny Grewal Daumerie[3], Neena Valecha[1]

[1]National Institute of Malaria Research, Dwarka, New Delhi, India; [2]National Vector Borne Disease Control Programme (NVBDCP), Odisha, India; [3]Medicines for Malaria Venture, Geneva, Switzerland.

*Corresponding author: Dr Anupkumar R. Anvikar

Contents

Figure S1. Study sites in Odisha State, India 2

Figure S2. Visual tool used by Accredited Social Health Activists (ASHAs) to recognize and report hemolytic symptoms. 3

Figure S3. The proportion of patients with Hb levels equal or greater than baseline levels (day 0) at each study follow-up day. 4

Figure S4. Individual patient hemoglobin declines from baseline at the nadir (post-hoc analysis). Bars are mean ± standard deviation. 5

Figure S5. Time course of hemoglobin (Hb) levels for patients with a ≥ 2 g/dL decrease in Hb versus baseline (day 0) at any point during the study by G6PD phenotype (qualitative testing). G6PDd was confirmed as G6PD Orissa (heterozygous) by genotyping. 6

Figure S1. Study sites in Odisha State, India.

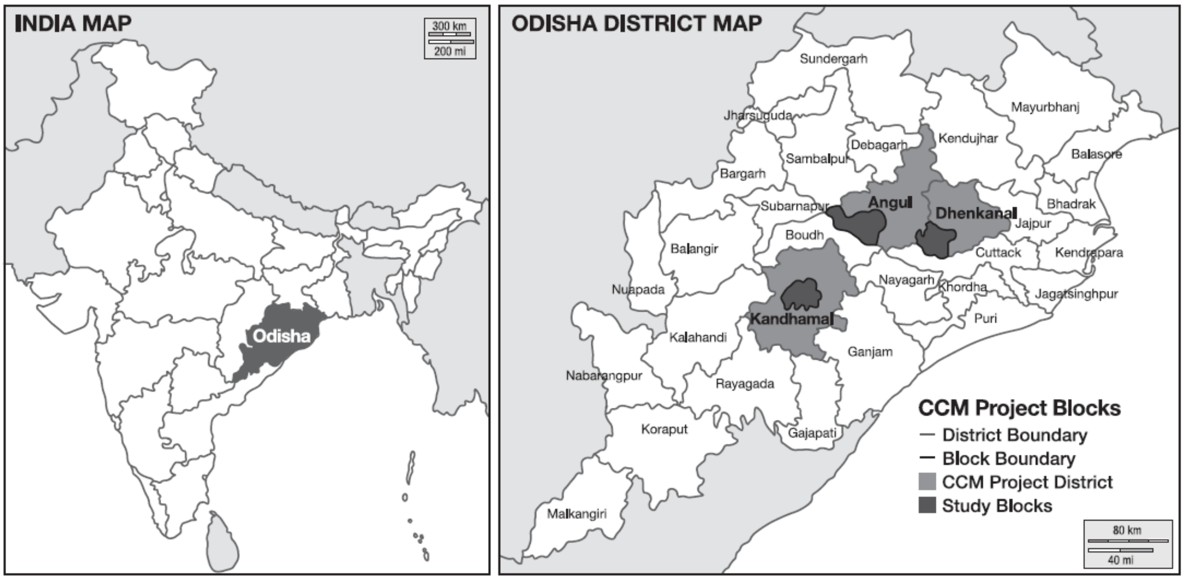

Figure S2. Visual tool used by Accredited Social Health Activists (ASHAs) to recognize and report hemolytic symptoms.

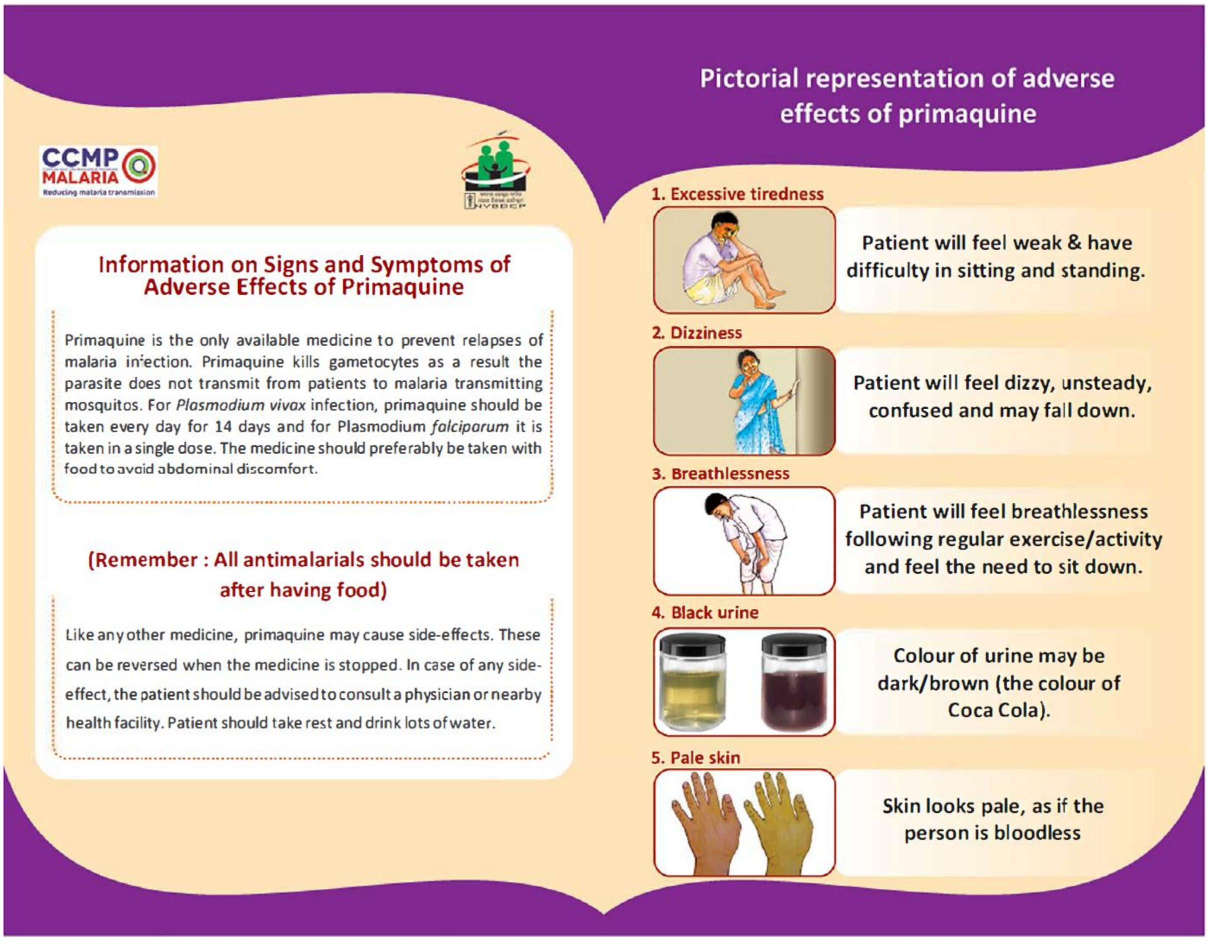

Figure S3. The proportion of patients with Hb levels equal or greater than baseline levels (day 0) at each study follow-up day.

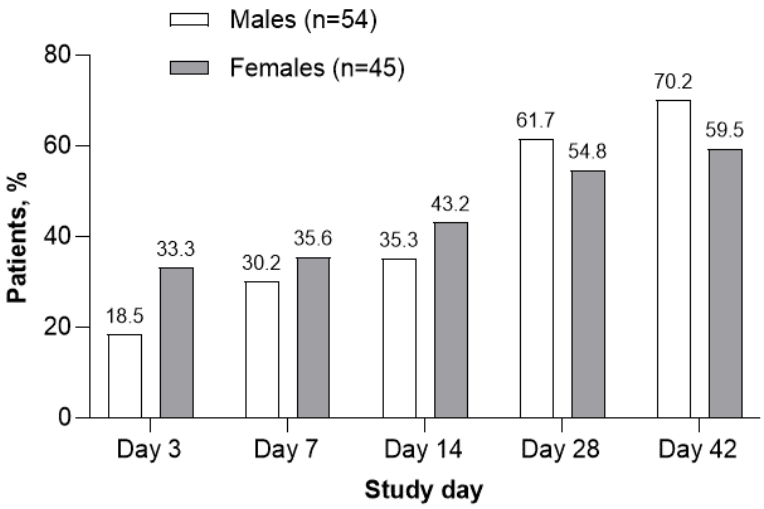

Figure S4. Individual patient hemoglobin declines from baseline at the nadir (post-hoc analysis). Bars are mean ± standard deviation.

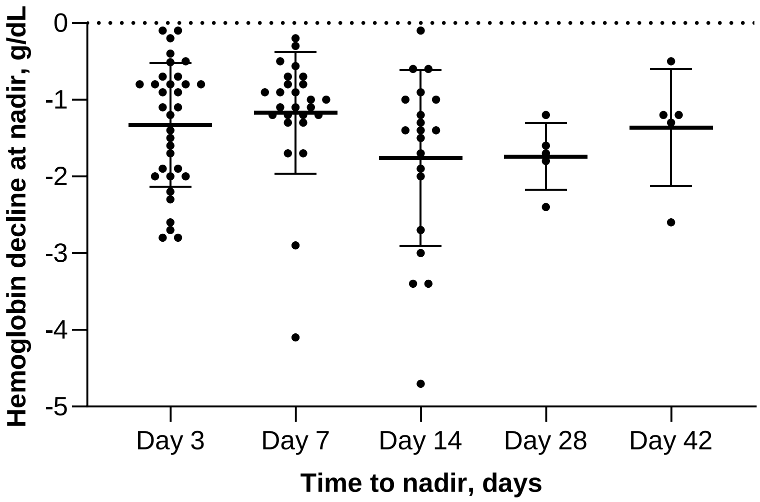

Figure S5. Time course of hemoglobin (Hb) levels for patients with a ≥ 2 g/dL decrease in Hb versus baseline (day 0) at any point during the study by G6PD phenotype (qualitative testing). G6PDd was confirmed as G6PD Orissa (heterozygous) by genotyping.

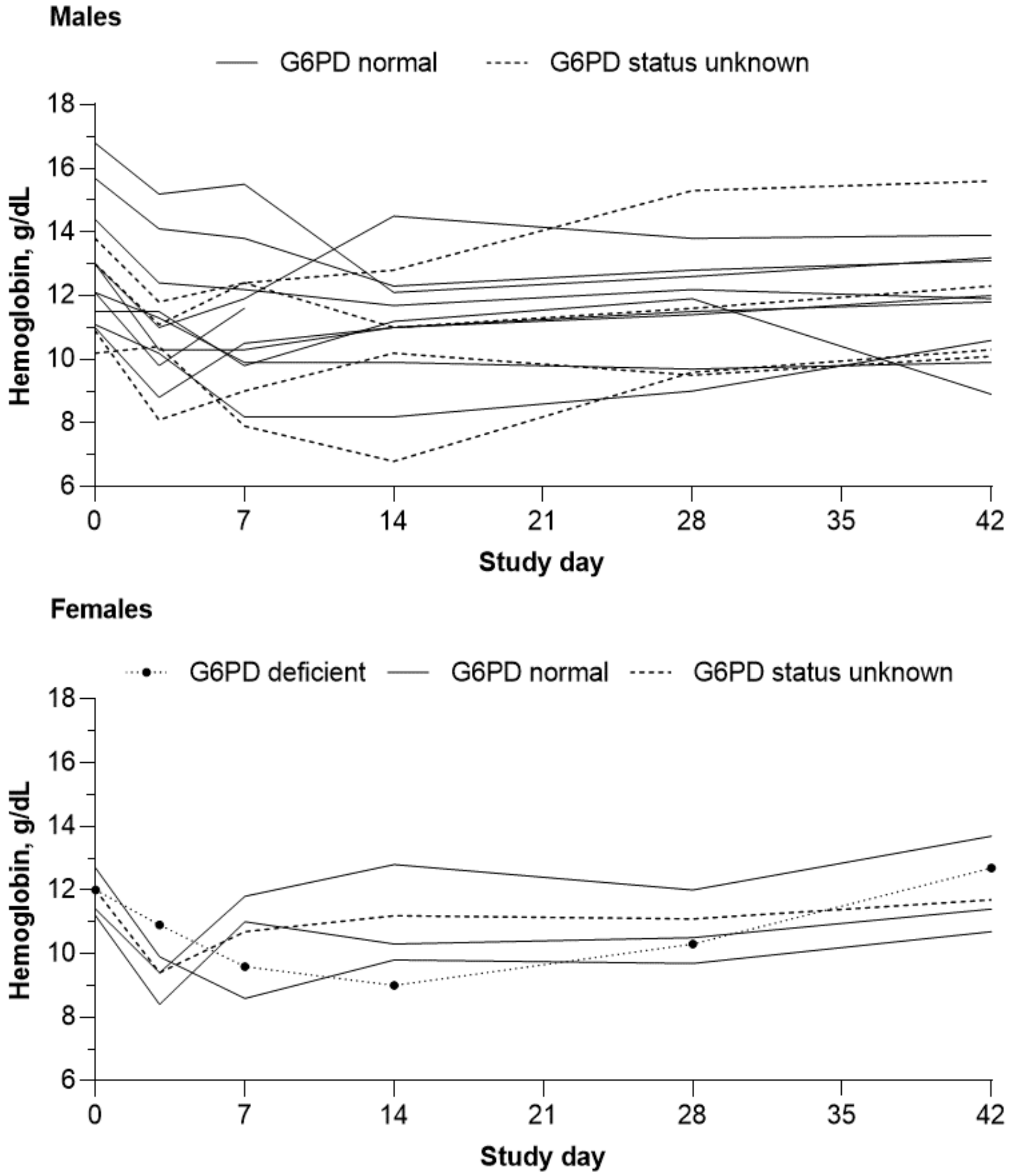

Supplement: Supplementary file 1 [file tpmd210816.SD1.pdf]
